# Features of randomised trials designed by the NPEU Perinatal Trials Service during Adrian Grant’s directorship

**DOI:** 10.1186/s12978-018-0567-7

**Published:** 2018-07-09

**Authors:** Diana Elbourne

**Affiliations:** Healthcare Evaluation, Department of Medical Statistics, London School of Hygiene & Tropical Medicine, Keppel Street, London, WC1E 7HT UK

## Abstract

Adrian Grant pioneered methodological innovations in the randomised trials organised by the Perinatal Trials Service established at the national Perinatal Epidemiology Unit in Oxford, UK. This Commentary discusses these innovations, and shows the wide range of trials designed under his directorship.

## Background

In his article recording Adrian Grant’s pioneering use of evidence synthesis in perinatal medicine between 1980 and 1992, Iain Chalmers [1] quoted from a 1984 letter published in The Lancet which Iain and I had co-authored with Adrian [2]. In that letter we alluded to some of the additional principles - beyond the need for systematic review of existing evidence - which became methodological features of the wide range of randomised trials organised by the Perinatal Trials Service (PTS) established at the national Perinatal Epidemiology Unit (NPEU). The trials are listed in Appendix 1.

## Methodological features

### Appropriate size

Our 1984 letter concluded with a warning that, to avoid the dangers of false inferences from non-randomised comparisons and small randomised trials, many perinatal controlled trials require sample sizes larger than any single unit can generate within a reasonable length of time. Although one centre was sufficient to obtain sufficient sample sizes to address some questions, for other questions, multicentre (often international) trials were needed.

### Secure, random allocation

The NPEU PTS used a variety of contextually appropriate methods for secure random allocation - sequentially numbered sealed opaque envelopes, sequentially numbered drug vials, and central random allocation when there was sufficient time to make a call and where reasonable telecommunications existed.

### Appropriate design

Most of the trials used two-armed, individually randomised designs. Where appropriate, more complex designs were employed, including factorial trials and a cluster randomised trial.

### Involving the views of care-givers and patients and their families

The PTS recognised that our work needed to address questions considered important by caregivers and families, so they were involved in deciding which questions to address, trial design and conduct, and dissemination of results. Taking account of families meant that many PTS trials investigated long term outcomes, such as pain, dyspareunia and incontinence for women, and disability for children.

### Facilitating infrastructure

A programme of randomised trials to support these methodological underpinnings needed an infrastructure and the PTS was established in 1982 “to provide a service to busy clinicians who wish to mount large simple-in-design randomized trials...[aiming] to identify moderate, but clinically useful, effects of promising treatments for the most important problems in perinatal care” [3].

The PTS had a flexible five-person core staff and others employed to work on specific trials. This continuity of staff enabled us to build standard operating systems. International trials needed particularly careful coordination, and the provision of trials materials in a number of languages. The eclampsia trial [4], for example was preceded by a pilot study in Argentina, with materials in Spanish.

## Adrian Grant’s legacy for perinatal trials

Over the years that Adrian Grant designed perinatal trials in the NPEU, the above methodological innovations and others are listed in the Table 1 below.

**Table 1:** Adrian Grant’s methodological innovations

• Identification and prioritisation of important questions	
• Systematic reviews	
• Alliance of patients, carers, and clinicians	
• Efficient trial conduct	
• Secure randomisation	
• Appropriate design, outcomes, and size	
• Support for participants	
• Newsletters	
• Integral economic evaluation	
• Embedded methodological research	
• Long term follow up	
• Feedback of trial results	

Adrian continued to support trials in Aberdeen after his move to direct the Health Services Research Unit there (https://www.abdn.ac.uk/hsru) in 1994, and then for the National Institute for Health Research (http://www.nihr.ac.uk).

## Conclusions

Many people are grateful for Adrian’s methodological rigor, his innovative approaches, and his generosity of support, mentoring and teaching. The lives of many babies and their families have been improved by Adrian’s pioneering work in perinatal trials, and the PTS that Adrian created has gone on to become a highly successful Clinical Trials Unit (https://www.npeu.ox.ac.uk/ctu).

## Appendix

### Randomised trials designed by the NPEU Perinatal Trials Service during Adrian Grant’s directorship

**Table 2:** Antenatal interventions

• Chorion villus sampling vs amniocentesis [5]	
• Cervical cerclage [6, 7]	
• Breast shells and Hoffman’s exercises [8]	
• Formal fetal movement counting [9]	
• Placental grading by ultrasonography [10]	
• Anti-convulsants for eclampsia [4]	
• Low dose aspirin [11, 12]	
• Fish-oil supplementation [13]	
• ‘Know your Midwife’ [14]	
• Social support [15]	

**Table 3:** Intrapartum interventions

• Dublin intrapartum fetal heart rate monitoring [16–19]	
• Vacuum extraction vs forceps (Portsmouth operative delivery) [20–22]	
• Vacuum extraction: different cups [23]	
• Fetal scalp electrode [24]	
• Perineal management (Berkshire) [25, 26]	
• Perineal suture (Southmead) [27]	
• Catgut for the repair of perineal trauma [28, 29]	
• Ipswich perineal repair [30–33]	

**Table 4:** Postnatal interventions

• Pelvic floor exercises [34]	
• Salt and Savlon bath concentrate [35]	
• Ultrasound and pulsed electromagnetic energy treatment for perineal trauma [36, 37]	

**Table 5:** Neonatal interventions

• Neonatal ventriculomegaly [38, 39]	
• Dexamethasone [40, 41]	
• Prophylactic ethamsylate [42, 43]	
• Surfactant [44]	
• Extracorporeal membrane oxygenation [45–53]	
